# Sarcopenic Obesity and Endocrinal Adaptation with Age

**DOI:** 10.1155/2013/204164

**Published:** 2013-04-11

**Authors:** Kunihiro Sakuma, Akihiko Yamaguchi

**Affiliations:** ^1^Research Center for Physical Fitness, Sports and Health, Toyohashi University of Technology, 1-1 Hibarigaoka, Tenpaku-cho, Toyohashi 441-8580, Japan; ^2^School of Dentistry, Health Sciences University of Hokkaido, Kanazawa, Ishikari-Tobetsu, Hokkaido 061-0293, Japan

## Abstract

In normal aging, changes in the body composition occur that result in a shift toward decreased muscle mass and increased fat mass. The loss of muscle mass that occurs with aging is termed sarcopenia and is an important cause of frailty, disability, and loss of independence in older adults. Age-related changes in the body composition as well as the increased prevalence of obesity determine a combination of excess weight and reduced muscle mass or strength, recently defined as sarcopenic obesity. Weight gain increases total/abdominal fat, which, in turn, elicits inflammation and fatty infiltration in muscle. Sarcopenic obesity appears to be linked with the upregulation of TNF-*α*, interleukin (IL)-6, leptin, and myostatin and the downregulation of adiponectin and IL-15. Multiple combined exercise and mild caloric restriction markedly attenuate the symptoms of sarcopenic obesity. Intriguingly, the inhibition of myostatin induced by gene manipulation or neutralizing antibody ameliorates sarcopenic obesity via increased skeletal muscle mass and improved glucose homeostasis. In this review, we describe the possible influence of endocrinal changes with age on sarcopenic obesity.

## 1. Introduction 

 Skeletal muscle contractions power human body movements and are essential for maintaining stability. Skeletal muscle tissue accounts for almost half of the human body mass and, in addition to its power-generating role, is a crucial factor in maintaining homeostasis. Given its central role in human mobility and metabolic function, any deterioration in the contractile, material, and metabolic properties of skeletal muscle has an extremely important effect on human health. Aging is associated with a progressive decline of muscle mass, quality, and strength, a condition known as sarcopenia [1]. The term sarcopenia, coined by I. H. Rosenberg, originates from the Greek words *sarx* (flesh) and *penia* (loss). Although this term is applied clinically to denote loss of muscle mass, it is often used to describe both a set of cellular processes (denervation, mitochondrial dysfunction, inflammatory, and hormonal changes) and a set of outcomes such as decreased muscle strength, decreased mobility and function [2], increased fatigue, a greater risk of falls [3], and reduced energy needs [4]. In addition, reduced muscle mass in aged individuals has been associated with decreased survival rates following critical illness [5]. In most countries, there has been a rapid and continuing increase in life expectancy. By the year 2030, 20% of the adult USA population will be older than 65 years [6]. In the 27 member states of the EU, the percentage of people aged 65 years and older will rise from 17.1 in 2008 to 25.4 in 2035 and to 30 in 2060 [7]. The estimated direct healthcare costs attributable to sarcopenia in the USA in 2000 were $18.5 billion ($10.8 billion in men and $7.7 billion in women), which represented about 1.5% of total healthcare expenditures for that year [8]. Therefore, age-related losses in skeletal muscle mass and function present an extremely important current and future public health issue. 

 Lean muscle mass generally contributes up to ~50% of total body weight in young adults but declines with aging to be 25% at 75–80 years old [9, 10]. The loss of muscle mass is typically offset by gains in fat mass. The loss of muscle mass is most notable in the lower limb muscle groups, with the cross-sectional area of the vastus lateralis being reduced by as much as 40% between the age of 20 and 80 years [11]. On a muscle fiber level, sarcopenia is characterized by specific type II muscle fiber atrophy, fiber necrosis, and fiber-type grouping [11–13]. In elderly men, Verdijk et al. [13] showed a reduction in type II muscle fiber satellite cell content with aging. Although various investigators showed very contradicting results for age-dependent changes of satellite cell numbers [13–16], most studies point to an age-dependent reduction in muscle regenerative capacity due to reduced satellite cell proliferation and differentiation. 

 Another morphologic aspect of sarcopenia is the infiltration of muscle tissue components by lipids because of the increased frequency of adipocyte or lipid deposition [17, 18] within muscle fibers. As with precursor cells in bone marrow, liver, and kidney, muscle satellite cells that can express an adipocytic phenotype increase with age [19], although this process is still relatively poorly understood in terms of its extent and spatial distribution. Lipid deposition, often referred to as intramyocellular lipid, may result from a net buildup of lipids due to the reduced oxidative capacity of muscle fibers with aging [17, 20].

 Several possible mechanisms for age-related muscle atrophy have been described; however, the precise contribution of each is unknown. Age-related muscle loss is a result of reductions in the size and number of muscle fibers [21] possibly due to a multifactorial process that involves physical activity, nutritional intake, oxidative stress, and hormonal changes [3, 22]. The specific contribution of each of these factors is unknown, but there is emerging evidence that the disruption of several positive regulators (Akt and serum response factor) of muscle hypertrophy with age is an important feature in the progression of sarcopenia [23, 24].

 Obesity is currently epidemic in the USA, with almost 70% of Americans overweight and one of three obese [25]. Obesity is associated with increased morbidity and mortality, and there is unchallenged evidence that obesity increases the risk for the development of hypertension, dyslipidemia, type 2 diabetes mellitus, sleep apnea, cancers of the breast, prostate, and colon, and all-cause mortality [26–28]. This review introduces the relationship between endocrinal changes with age and sarcopenic obesity. 

## 2. Sarcopenic Obesity

 Aging is associated with important changes in body composition and metabolism [29, 30]. Between the age of 20 and 70 years, there is a progressive decrease of fat-free mass (mainly muscle) of about 40% and a rise in fat mass. There is a relatively greater decrease in peripheral compared to central fat-free mass. After the age of 70 years, fat-free mass and fat mass decrease in parallel. Fat distribution changes with age such that there is an increase in visceral fat, which is more marked in women than in men. Also, fat is increasingly deposited in skeletal muscle and in the liver. The higher visceral fat is the main determinant of impaired glucose tolerance in the elderly. Increased intramuscular and intrahepatic fat contribute to impaired insulin action through locally released adipokines and fat-free fatty acids. Increased pancreatic fat with declining *β*-cell function also plays a role [31].

 Due to the loss of skeletal muscle, the basal metabolic rate declines by 2%-3% per decade after the age of 20 years, by 4% per decade after the age of 50 years, equating approximately 150 kcal per day, and overall by 30% between the age of 20 and 70 years [32]. This, together with decreased intensity and duration of physical activity as well as decreased postprandial energy expenditure due to a decreased fat oxidation, accounts for the decreased energy expenditure seen with aging. Medical complications of obesity in the elderly are mainly concentrated around the metabolic syndrome (with glucose intolerance, hypertension, dyslipidaemia, and cardiovascular disease). The metabolic syndrome peaks at the age of 50–70 years in males and of 60–80 years [33]. The metabolic syndrome is a recognized risk factor for strole but is also related to subclinical ischaemic brain lesions, placing the subjects at risk for future cognitive impairment [34]. Obesity also increases the risk of heart failure, and estimates suggest that having a body mass index (BMI) > 30 kg/m^2^ doubles the risk [35]. Other obesity-related disorders are osteoarthritis, pulmonary dysfunction such as the obstructive sleep apnoea syndrome, certain cancer types, reduced cognitive skills, and urinary incontinence [6, 36, 37]. 

 The obesity elderly are also likely to have functional limitations because of the decreased muscle mass and strength and increased join dysfunction, disabilities of activities of daily living, frailty, chronic pain, and impaired quality of life [6, 38]. Indeed, Baumgartner [39] observed that men and women older than 60 years of age with sarcopenic obesity showed, respectively, an 8- and 11-fold higher risk of having three or more physical disabilities. More importantly, it was observed that the association with functional status impairment was stronger for sarcopenic obesity than for either obesity or sarcopenia alone. Unintentional injuries such as sprains and strains occur more often [40]. Obesity is an important risk for frailty either through increased levels of inflammatory markers or through sarcopenia [41].

 Interestingly, the proposed mechanism involved in sarcopenic obesity could be the increased production from adipose tissue of different substances, such as tumor necrosis factor-*α* (TNF-*α*) and leptin, which are known to influence insulin resistance and growth hormone (GH) secretion [42]. This hypothesis has been confirmed by Schrager et al. [43] who observed in a large-scale sample of men and women that the degree of obesity, as evaluated by BMI and its distribution, and by waist circumference, directly affected inflammation which in turn contributed to the development and progression of sarcopenia. Further increases in leptin, at least partially depending on the age-related fat mass increase, may lead to leptin resistance and thus to a reduction of fatty acid oxidation in muscles, contributing to ectopic fat deposition in organs such as the liver, heart, and muscles [44] and, in turn, to the loss of muscle quality in obese older subjects. 

 Studies in both humans and animals demonstrate that obesity is a state of low-grade, chronic inflammation, characterized by elevated circulating proinflammatory molecules produced predominantly from enlarged adipocytes and activated macrophages in adipose tissue [45, 46]. Lipocalin-2 would be a possible candidate regulating the amount of adipose tissue under chronic inflammation and insulin resistance. Lipocalin-2 is abundantly produced by adipocytes [47, 48]. Expression of lipocalin-2 in adipose tissue is elevated in various experimental models of obesity and in obese humans [49–51]. Its expression can be induced by various inflammatory stimuli, including lipopolysaccharides and interleukin (IL)-1*β* [52, 53]. Intriguingly, lipocalin-2 deficiency in mice elicits marked decreases in the expression and the activity of 12-lipoxygenase, an enzyme responsible for metabolizing arachidonic acid, and the production of TNF-*α*, a critical insulin resistance-inducing factor [54]. It remains to be elucidated whether lipocalin-2 levels increase with normal aging and further with sarcopenic obesity in mammals. 

## 3. Endocrinal Adaptation with Age

### 3.1. GH and Testosterone

 Testosterone increases muscle protein synthesis [55], and its effects on muscle are modulated by several factors including genetic background, nutrition, and exercise [56]. In males, levels of testosterone decrease by 1% per year and those of bioavailable testosterone by 2% per year from age 30 [57, 58]. In women, testosterone levels drop rapidly from 20 to 45 years of age [59].

 GH is a single-chain peptide of 191 amino acids produced and secreted mainly by the somatotrophs of the anterior pituitary gland. GH coordinates the postnatal growth of multiple target tissues, including skeletal muscle [60]. GH secretion occurs in a pulsatile manner with a major surge at the onset of a slow-wave sleep and less conspicuous secretory episodes a few hours after meals [61]. The secretion of GH is maximal at puberty accompanied by very high circulating insulin-like growth factor-I (IGF-I) levels [62], with a gradual decline during adulthood. Indeed, circulating GH levels decline progressively after 30 years of age at a rate of ~1% per year [63]. In aged men, daily GH secretion is 5- to 20-fold lower than that in young adults [64]. Therefore, many researchers have indicated age-related endocrine defects such as decreases in anabolic hormones. Although hormonal supplementation for the elderly has been conducted on a large scale, it was found not to be effective against sarcopenia and to have minor side effects [64–67].

 Increased adiposity is often associated with high circulating levels of free fatty acids [68, 69], which inhibit GH production and decrease plasma levels of IGF-I [70, 71]. A recent study showed that sarcopenic obese persons had depressed GH secretion compared to obese persons [72]. Similarly, obese individuals tend to have lower testosterone levels [73]. Of note, low levels of these anabolic hormones have been reported to be positively associated with low muscle strength [74, 75] and may therefore contribute to muscle impairment in obese individuals [76]. 

### 3.2. Insulin

 Insulin is a powerful anabolic signal in proteins [77]. Insulin was infused directly into the femoral artery to increase the leg insulin levels to approximate postprandial values while avoiding systemic hypoaminoacidemia. Insulin significantly stimulated muscle protein synthesis in young but not older subjects. There was no significant change in muscle protein breakdown as measured by two- and three-pool modeling. The increase in synthesis in young subjects resulted in a shift from a negative to positive protein net balance across the leg-indicating overall net protein accretion during the clamp in young subjects. In the older subjects, however, the net muscle protein balance remained negative. Insulin resistance has been long recognized as a characteristic of aging in humans and rodents [78]. Blood flow was lower in older as compared to younger subjects at baseline and during the clamp and tended to increase from baseline in young adults only during the clamp. As hypothesized by  Timmerman and Volpi [79], this effect was likely mediated through insulin-induced vasodilation. Insulin is a potent stimulator of the endothelial-derived vasodilator and nitric oxide [80]. In a subsequent study, they reported that this age-related insulin resistance of muscle protein synthesis could be overcome by increasing insulin levels to approximately double the postprandial levels via improvements in mammalian target of rapamycin signaling [81]. 

 Available experimental evidence points to the development of adiposity as the main cause of the decreased insulin action in old rats [82] and elderly humans [83, 84]. Studies in rats have demonstrated that fat mass accretion occurs at early aging and is paralleled by a marked decrease of insulin action in visceral fat tissue. 

### 3.3. TNF-*α*, IL-6, and C-Reactive Protein (CRP)

 Inflammation may negatively influence skeletal muscle through direct catabolic effects or through indirect mechanisms (i.e., decreases in GH and IGF-I concentrations, induction of anorexia, etc.) [85]. There is growing evidence that higher levels of inflammatory markers are associated with physical decline in older individuals, possibly through the catabolic effects of these markers on muscle. In an observational study of more than 2000 men and women, TNF-*α* showed a consistent association with declines in muscle mass and strength [86]. The impact of inflammation on the development of sarcopenia is further supported by a recently published animal study showing that a reduction in low-grade inflammation by ibuprofen in old (20 months) animals resulted in a significant decrease in muscle mass loss [87]. An age-related disruption of the intracellular redox balance appears to be a primary causal factor for a chronic state of low-grade inflammation. More recently, Chung et al. [88] hypothesized that abundant nuclear factor-*κ*B (NF-*κ*B) protein-induced age-related increases in IL-6 and TNF-*α*. Moreover, reactive oxygen species (ROS) also appear to function as second messengers for TNF-*α* in skeletal muscle, activating NF-*κ*B either directly or indirectly [89]. Indeed, marked production of ROS has been documented in muscle of the elderly [90, 91]. However, it is not clear whether NF-*κ*B signaling is enhanced with age. Despite some evidence supporting enhanced NF-*κ*B signaling in type I fibers of aged skeletal muscle, direct evidence for increased activation and DNA binding of NF-*κ*B is lacking [92, 93]. For example, Philips and Leeuwenburgh [93] found that neither p65 protein expression nor the binding activity of NF-*κ*B was significantly altered in the vastus lateralis muscles of 26-month-old rats despite the marked upregulation of TNF-*α* expression in both blood and muscle. Upregulated TNF-*α* expression in serum and muscle seems to enhance apoptosis in mitochondria resulting in a loss of muscle fibers [93–95]. It has been shown that TNF-*α* is one of the primary signals inducing apoptosis in muscle. 

 IL-6 and CRP, known as “geriatric cytokines”, are multifunctional cytokine produced in situations of trauma, stress, and infection. During the aging process, levels of both IL-6 and CRP in plasma become elevated. The natural production of cytokines is likely beneficial during inflammation, but the overproduction and the maintaining of an inflammatory state for long periods of time, as seen in elderly individuals, is detrimental [96, 97]. A number of authors have demonstrated that a rise in plasma levels of proinflammatory cytokines, especially IL-6, and proteins under acute conditions is associated with a reduction in mobility as well as a reduced capacity to perform daily activities, the development of fragility syndrome, and increased mortality rates [96–98]. In older men and women, higher levels of IL-6 and CRP were associated with a two- to three-fold greater risk of losing more than 40% of grip strength over 3 years [99]. In contrast, there were no longitudinal associations between inflammatory markers and changes in grip strength among high functioning elderly participants from the MacArthur Study of Successful Aging [100]. More recently, Hamer and Molloy [101] demonstrated, in a large representative community-based cohort of older adults (1,926 men and 2,260 women (aged 65.3 ± 9.0 years)), that CRP was associated with poorer hand grip strength and chair stand performance in women but only chair stand performance in men. In addition, Haddad et al. [102] demonstrated atrophy in the tibialis anterior muscle of mice following the injection of relatively low doses of IL-6. In a recent randomized trial that employed aerobic and strength training in a group of elderly participants, significant reductions in various inflammatory markers (IL-6, CRP, and IL-18) were observed for aerobic but not strength training [103]. In contrast, combined resistance and aerobic training that increased strength by 38% resulted in significant reductions in CRP [104]. 

### 3.4. Myostatin

 Myostatin was first discovered during screening for novel members of the transforming growth factor-*β* superfamily and shown to be a potent negative regulator of muscle growth [105]. Mutations in myostatin can lead to massive hypertrophy and/or hyperplasia in developing animals, as evidenced by knockout experiments in mice. Myostatin levels increase with muscle atrophy due to unloading in mice and humans [106, 107] and with severe muscle wasting in HIV patients [108]. Administration of myostatin *in vivo* to adult mice induces profound muscle loss analogous to that seen in human cachexia syndromes [109]. Together, these studies suggest that increased levels of myostatin lead to muscle wasting. 

 Many researchers have conducted experiments to inhibit myostatin in models of muscle disorders such as Duchenne muscular dystrophy, ALS, and cancer cachexia [23]. In addition, several investigators examined the effect of inhibiting myostatin to counteract sarcopenia using animals [110, 111]. More recently, Murphy et al. [111] showed, by way of one-weekly injections, that a lower dose of PF-354 (10 mg/Kg) significantly increased the fiber cross-sectional area (by 12%) and *in situ* muscle force (by 35%) of aged mice.

 Skeletal muscle is the primary site of insulin-mediated glucose disposal, the largest reservoir of glycogen in the human body, and a key determinant of energy expenditure. Hence, several recent studies have also investigated the effects of genetic and pharmacological inhibition of myostatin, and the resultant resistance-trained phenotype, on the prevention and treatment of obesity and type 2 diabetes mellitus [112, 113]. Similar to these results, Zhang et al. [114] demonstrated that the inhibition of myostatin increased skeletal muscle mass and reduced body weight, fat mass, and circulating concentrations of triacylglycerol caused by a high-fat diet. Postnatal blockade of myostatin with a neutralizing antibody in obese insulin-resistant mice significantly improved glucose homeostasis, lowered circulating triacylglycerols, and increased circulating concentrations of the adipose tissue-derived cytokine and adiponectin [115, 116]. These findings highlight the therapeutic potential of antibody-directed myostatin inhibition for sarcopenic obesity. Although many researchers expect myostatin levels to be increased not only in muscle but also in serum, blood myostatin levels have not been shown to increase with age [117].

### 3.5. Adiponectin and Leptin

 Adipose tissue itself generates a myriad of hormones and other bioactive proteins, including leptin (in normal concentrations induces satiety and regulates body composition) and adiponectin (anti-inflammatory and antiatherogenic) [118]. Adiponectin is an abundant plasma protein. Structurally, adiponectin contains a carboxyl-terminal globular domain and an amino-terminal collagenous domain and also shares extensive sequence homology with collagen VIII and X [119]. Adiponectin circulates in serum as a range of multimers from low-molecular weight trimers to high-molecular weight dodecamers [120]. With the exception of severe cases of undernutrition [121] and in the newborn [122], there is a strong negative correlation between plasma adiponectin concentrations in humans and fat mass [119], with obesity reducing adiponectin levels and weight reduction increasing them [45, 123]. 

 Adiponectin has been shown to improve a whole-body insulin sensitivity in models of genetic and diet-induced obesity [124, 125]. Adiponectin stimulates fatty acid oxidation and glucose uptake in skeletal muscle [126] and adipose tissue [127], effects which are dependent on AMP-activated protein kinase (AMPK) signaling. The activation of adiponectin is dependent on signaling through adiponectin receptor AdipoR1 and AdipoR2. A study in human skeletal muscle [128] and in primary myotubes [129] suggested that skeletal muscle contains abundant levels of both AdipoR1 and AdipoR2 but that liver primarily expresses AdipoR2. Adiponectin's activation of AMPK signaling is blunted in obesity [130], despite similar AdipoR1 and AdipoR2 expression. Adiponectinlevels also decline with age [131]. Adiponectin activates AMPK and inhibits NF-*κ*B signaling, decreasing monocyte, macrophage, and dendritic cell production of TNF-*α* and interferon (IFN)-*γ* while increasing the production of anti-inflammatory cytokines, IL-10, and IL-1R*α* [45]. Adiponectin directly inhibits natural killer (NK) cells by preventing IL-2-stimulated cytotoxicity and IFN-*γ* production [132].

 In contrast to adiponectin levels, serum leptin levels reflect overall adipose mass [45]. Leptin is an adipokine that regulates energy balance and glucose homeostasis [133]. Leptin acts mainly through the central nervous system, binding to specific hypothalamic receptors and regulating appetite, neuroendocrine pathways, and the autonomic nerves which bring about effects on peripheral tissues [134]. Nevertheless, leptin receptor expression has been reported to occur in pancreatic *β*-cells, muscle, liver, and fat, among other peripheral tissues, suggesting the existence of a direct effect of leptin in addition to its central action [135]. With the exception of fat tissue [136, 137], *in vivo* treatment with leptin has an insulin-sensitizing effect on peripheral tissue. In skeletal muscle, chronic peripheral leptin administration induces an increase of glucose uptake under euglycemic- hyperinsulinemic conditions [137, 138], and the same has been observed after the microinjection of leptin into the ventromedial hypothalamus [136]. In addition, leptin is largely proinflammatory because leptin increases TNF-*α*, IL-6, and IL-12 production by monocytes [45, 118]. Serum leptin levels and hypothalamic leptin resistance increase with age [139].

 Interestingly, in obese but not in lean rats, leptin administration has been proven to decrease insulin signaling in liver [140]. Since obese rats show central leptin resistance and hyperleptinemia similar to aged rats [141], it can be speculated that during aging, the direct effects of leptin on peripheral tissues could prevail over its central action and contribute to the development and maintaining of a state of insulin resistance.

### 3.6. IL-10 and IL-15

 Serum IL-10 may be positively correlated with obesity in middle aged humans [142]. Exercise releases IL-10 into the circulation, implying production by skeletal muscle [143]. Macrophage IL-10 production increases in old mice [144, 145]. Two recent studies showed marked increase in serum IL-10 in elderly humans [146], although an earlier study did not show a significant difference between middle-aged and very old humans [147]. IL-10 is broadly anti-inflammatory, inhibiting antigen presentation and suppressing release of TNF-*α*, IL-2, IFN-*γ*, IL-4, and other cytokines [148]. Indeed, mice homozygous for targeted deletion of the IL-10 gene had elevated levels of TNF-*α*, IL-6, IFN-*γ*, and IL-1*β* in serum particularly at a later age (between 72 and 90 weeks) [149]. In addition, these mice had higher mortality rates when compared to age and sex-matched B6 control mice. On the other hand, IL-10 stimulated NK cell proliferation, cytotoxicity, and cytokine secretion *in vitro* when combined with IL-1 [150]. In murine cytomegalovirus-infected mice, IL-10 promoted NK cell cytotoxic granule release but increased NK cell activation-induced cell death [151]. In the elderly cohort, BMI correlated inversely with the percentage of NK cells and correlated directly with the NK cell apoptosis rate [152]. Therefore, serum IL-10 levels may regulate the amount of adipose tissue by modulating several inflammatory cytokines and/or recruiting immune cells (e.g., NK cells).

**Figure 1:**
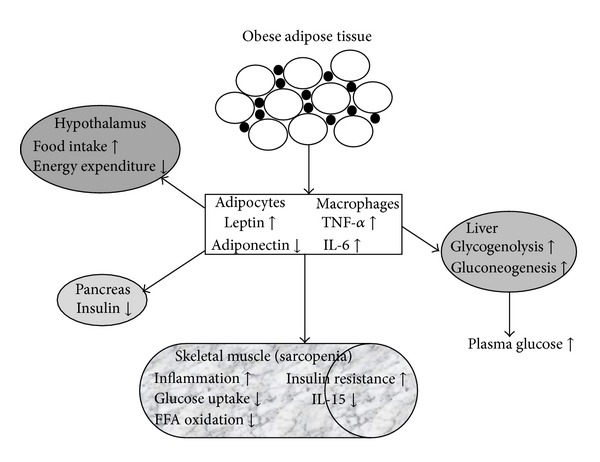
Obesity-induced changes in adipokine secretion and the development of insulin resistance in sarcopenic muscle. Expansion of adipose tissue in obesity leads to increased macrophage infiltration and inflammation with enhanced production of proinflammatory cytokines such as TNF-*α* and IL-6. This is accompanied by a dysregulated secretion of leptin and adiponectin. These adipocyte- and macrophage-derived adipokines elicit a variety of adverse effects on numerous tissues including the hypothalamus, liver, pancreas, and skeletal muscle. On the systemic level, altered adipokine secretion can lead to increased food intake and reduced energy expenditure through actions in the hypothalamus and to decreased muscle insulin sensitivity.

 IL-15 mRNA is expressed in many tissues [153], but IL-15 biosynthesis is very complex, and RNA levels do not necessarily indicate protein secretion. IL-15 isoforms have alternative signal peptides of 21 and 48 amino acids. Importantly, IL-15 requires the presence of IL-15R*α* for efficient biosynthesis and secretion [154, 155]. Like IL-15, IL-15R*α* synthesis is widespread within and outside of lymphoid tissues. Skeletal muscle tissue produces very high levels of IL-15 and expresses IL-15R*α* [156]. IL-15 levels are reported to increase transiently immediately following resistance [157] and aerobic [158] exercise, suggesting that IL-15 is indeed released from muscle tissue. In mice, muscle and serum IL-15 protein levels decline progressively with advanced age [159]. A study of aging rats showed that a longevity-promoting regimen of calorie restriction prevented age-related declines in muscle IL-15 expression observed in ad libitum-fed rats [94]. In an intriguing brief report involving human subjects, Gangemi et al. [160] observed significantly elevated serum IL-15 levels in centenarians living independently, suggesting high expression of IL-15 conferred protection from both frailty and age-related disease. IL-15 also has important effects on adipose tissue. IL-15 inhibits adipocyte differentiation in culture and obese people have low-blood IL-15 levels [156, 161, 162]. IL-15-deficient mice become obese despite unaltered food consumption; IL-15 injections reversed both this obesity and diet-induced obesity, lowered glucose levels and increased insulin sensitivity [161, 163].  Figure 1 provides an overview of the action of dysregulated adipokines to various organs (e.g., hypothalamus and skeletal muscle) in sarcopenic obesity.

## 4. Therapeutic Application

### 4.1. Physical Exercise (Combination)

 Adipose tissue infiltration of skeletal muscle increases with age [164, 165]. Recent studies have demonstrated that mitochondrial damage occurs in obese individuals due to enhanced ROS and chronic inflammation caused by increased fatty acid load [166]. Specifically, in skeletal muscle, the expression of PGC-1*α* drives not only mitochondrial biogenesis and the establishment of oxidative myofibers but also vascularization [167]. It was found that a high-fat diet or fatty acid treatment caused a reduction in the expression of PGC-1*α* and other mitochondrial genes in skeletal muscle [168]. A recent study has also demonstrated that transgenic overexpression of PGC-1*α* in skeletal muscle improved sarcopenia and obesity associated with aging in mice [169]. Therefore, the well-known sarcopenia-attenuating effects of endurance training may be attributable to the protection against mitochondrial disorders (apoptosis, oxidative damage, etc.) caused by an increase in the production of PGC-1*α* [167].

 The American College of Sports Medicine recommends a multicomponent training exercise programme (strength, endurance, balance, and flexibility) to improve and maintain physical function in older adults [170]. Resistance exercise has been investigated as an approach to counteract sarcopenia by stimulating protein synthesis and cause muscle hypertrophy with increased muscle strength and with improved physical performance [171]. Endurance training improves aerobic capacity. Most of the studies had a multicomponent program of 90-min sessions per week, consisting of 15 min of balance training, 15 min of flexibility, 30 min of aerobic exercise, and 30 min of high-intensity resistance training.

 To study the impact of each exercise modality in more detail, Davidson et al. [172] randomized 60- to 80-year-old obese subjects into 4 groups: a control group, a group that had progressive resistance training, a group that performed aerobic exercise, and a group that combined progressive resistance training with aerobic exercise. After 6 months, body weight decreased by 0.6 kg in the resistance, by 2.8 kg in the aerobic, and by 2.3 kg in the combined exercise group. Abdominal fat and visceral fat decreased and endurance capacity improved significantly in the aerobic and combined exercise group. Skeletal muscle mass and muscle strength increased in the resistance and combined exercise groups only. Insulin resistance improved by 31% in the aerobic group and by 45% in the combined exercise group, whereas it did not change in the resistance training group. The combination of progressive resistance training and aerobic exercise is the optimal exercise strategy for simultaneous improvement of insulin resistance and functional limitations in the elderly. Aerobic exercise only is the second best choice.

### 4.2. Nutrition and Diet

 Diet-induced weight loss results in a decrease in both fat mass and fat-free mass and so could exacerbate the age-related loss of muscle mass and further impair physical function. Based on intensive research concerning sarcopenia and sarcopenic obesity, dietary guidelines were adjusted to prevent sarcopenic obesity and to guide the medical profession in managing weight loss in the presence of sarcopenic obesity [173, 174].

 In the treatment of subjects with, or at risk of, sarcopenic obesity, the energy deficit should be more moderate than usual (range of 200–750 kcal) with emphasis on a higher intake of proteins (up to 1.5 g/Kg) of high biological quality, ensuring adequate renal function. When restricting energy intake, protein intake must be maintained or even increased as dietary protein, and amino acids are the most effective means to slow down or prevent muscle protein catabolism. In particular, Leucine is an important mediator of the response to amino acids. It increases muscle protein synthesis by modulating the activation of mammalian target of rapamycin complex 1 and signaling components of translation initiation [175]. In order to optimize the anabolic response to ingested high-quality proteins, certain peculiarities of old age have to be taken into account [173]. In contrast to younger people, the elderly have a diminished anabolic response to proteins when they are coingested with carbohydrates. 

## 5. Conclusions and Perspectives

 Obesity is a major public health problem. The population is growing older, and the prevalence of obesity in the elderly is rising. Aging and obesity are two conditions that present an important part of health costs. The impact of sarcopenic obesity on physical, metabolic, and cardiovascular functions is becoming a primary concern amongst nutritionists, geriatricians, and public health officers. The etiopathogenesis of sarcopenic obesity is complex and multiple factors can interplay, including lifestyle, endocrine, and immunological factors [176, 177]. Decreased physical activity and energy expenditure with aging predispose to fat accumulation and fat redistribution but muscle loss. Sarcopenic obesity seems to be modulated by an age-related decrease in serum IL-15 and adiponectin and/or chronic inflammation (upregulation of TNF-*α*, IL-6, and myostatin).

 Lifestyle intervention should be the first step, and its effects have been extensively in the obese elderly. Multicomponent exercise includes flexibility training, aerobic exercise, and resistance training. Obesity and specifically sarcopenic obesity, in the elderly, are potentially preventable, and should be tackled from younger ages and also during major later-life transitions such as retirement. 

## Abbreviations

AdipoR: Adiponectin receptor
AMPK: AMP-activated protein kinase
BMI: Body mass index
CRP: C-reactive protein
GH: Growth hormone
IFN: Interferon
IGF-I: Insulin-like growth factor-I
IL: Interleukin
NF-*κ*B: Nuclear factor-kappa B
NK: Natural killer
PGC-1*α*: Peroxisome proliferator-activated receptor *γ* coactivator 1*α*
ROS: Reactive oxygen species
TNF-*α*: Tumor necrosis factor-*α*.
